# Bibliometric study of research and development for neglected diseases in the BRICS

**DOI:** 10.1186/s40249-016-0182-1

**Published:** 2016-09-06

**Authors:** Jing Bai, Wei Li, Yang-Mu Huang, Yan Guo

**Affiliations:** 1 School of Public Health, Peking University Health Science Center, Xueyuan Road 38, Haidian District, Beijing, 100191 China; 2 Peking University Health Science Library, Xueyuan Road 38, Haidian District, Beijing, 100191 China

**Keywords:** Neglected diseases, Research and development, BRICS, Bibliometric analysis

## Abstract

**Background:**

Large numbers of people are suffering from a group of diseases that mainly affect developing countries, as there are no available or affordable products for prevention or treatment. Research and development (R&D) for these diseases is still a low priority on the health agenda. Brazil, Russia, India, China and South Africa (BRICS) are quickly growing economies and having more and more positive impact on global health. Additionally, their R&D capacity is believed to be enhanced through decades of investment in education and life science research. The BRICS, as a group of emerging and developing countries, are expected to make greater contributions to solving the problem that mainly affects the entire developing countries community. However, there has been little research to provide a macroscopic overview of BRICS’ effort in R&D for neglected diseases. The aim of this study is to investigate scientific production in BRICS countries in this area and their main research hotspots.

**Methods:**

Global relevant literature was searched without time limits through PubMed and high yield countries were identified using GoPubMed. Literature up to the end of 2013 from the BRICS was obtained and high frequency words were extracted and clustered using Bibliography Item Co-occurrence Mining System 2.0 (BICOMS) and Graphical Clustering Toolkit 1.0 (gCLUTO).

**Results:**

In total, 32, 47, 51, 31 and 44 high frequency words from Brazil, Russia, India, China and South Africa respectively were extracted for clustering analysis. The clustering indicated that eight diseases were research hotspots in BRICS countries. India had the most extensive hotspots and Brazil came in second. The other three countries shared common research foci: helminthiasis, Human Immunodeficiency Virus infection and Acquired Immune Deficiency Syndrome (HIV/AIDS) and tuberculosis.

**Conclusions:**

Developed countries still make the majority of contributions to R&D on neglected diseases, but BRICS countries are playing a growing role. Instead of the “big three diseases” (HIV/AIDS, malaria and tuberculosis) recognized by WHO, the BRICS focus more on major causes of disease burden in their own countries. Disease burden and domestic policy, especially patent law, exert primary influence on the research focus.

**Electronic supplementary material:**

The online version of this article (doi:10.1186/s40249-016-0182-1) contains supplementary material, which is available to authorized users.

## Multilingual abstracts

Please see Additional file 1 for translations of the abstract into the five official working languages of the United Nations.

## Background

Due to the development of global economy and the increase in investment in health, many diseases have been effectively prevented or treated by new products. However, for a certain group of diseases, which threaten almost 1/6 of the world population, mostly from developing countries, is still a low priority in health systems. Some of these diseases lead to severe disability and some hinder children’s physical and brain development. Neither pandemic outbreak nor massive mortality will usually be caused by these diseases and thus they have been neglected when developing health agendas and budgets [1]. Moreover, there are no effective products for the prevention, diagnosis or treatment due to the lack of motivation for product research and development. The root cause for the lack of R&D is that these diseases primarily affect poor populations living in developing countries with tropical or subtropical climates. As to the developing countries, their capacity for R&D investment and purchasing power are limited. Meanwhile, developed countries with strong R&D capacity are not interested in these diseases as they are less rewarding due to the low market demand and the affected areas usually exist outside their countries’ borders. The group of diseases with common attributes with regard to R&D and disease burden is named “neglected diseases”. Global Funding of Innovation for Neglected Disease (G-FINDER) defines neglected diseases as “[diseases that] predominantly affect developing countries and for which products are needed but there is insufficient commercial pull to stimulate R&D”, which covers 14 kinds of diseases, such as kinoplasmosome, helminthiasis and dengue [2].

World Health Organization (WHO) and other institutions have conducted several activities in order to improve the R&D for neglected diseases. Developing countries, especially BRICS countries, are expected by the international community to play a greater role in responding to the diseases that mainly affect developing countries [3, 4]. With their strengthened economy, the BRICS did shoulder more responsibilities. China has surpassed Japan as the world’s second-largest economy in 2010. Brazil, India and Russia were the seventh, ninth and tenth respectively [5]. BRICS countries are more actively engaged in global health with increased foreign aid spending. The spending is still relatively small compared to overall spending by the US and Western European countries, but it has been increasing rapidly in recent years. From 2005 to 2010, Brazil’s assistance spending increased by about 20.4 % per year, India’s by about 10.8 %, China’s by around 23.9 %, and South Africa’s by around 8.0 %. Russia’s assistance increased substantially early in the same period and stabilized at around 450 million US dollars each year [6]. Additionally, some of the countries’ health R&D capacity and ability to address their own health issues improved [7]. It is believed that many countries affected by neglected diseases, such as Brazil and India, have the infrastructure to conduct neglected disease research with the benefit of their investment in education and health research.

Up to now, the BRICS’ contributions in R&D for neglected diseases are not clear because there is little macroscopic overview on it. Bibliometric analysis, a well-established research method in information science, has been commonly used for revealing research efforts. It is used for the quantitative description of documents in diseases, such as a group of diseases or a specific one [8, 9]. The five countries might differ from each other in R&D for neglected diseases due to the varied disease burden, health policy and investment. Bibliometric analysis can present the different landscapes of each country precisely. This study tries to find out what kind of contribution the BRICS are making in this area and the R&D focus for each country using cluster analysis of published articles. An empiric perspective on what the BRICS have studied in neglected diseases might inform groups making a joint effort in R&D for neglected diseases.

## Methods

PubMed, which is the most comprehensive medical literature database worldwide, was chosen as the English literature source. PubMed is a free resource that is developed and maintained by the National Center for Biotechnology Information at the U.S. National Library of Medicine, which includes the fields of biomedicine and health, covering portions of the life sciences, behavioral sciences, chemical sciences, and bioengineering. PubMed comprises of over 25 million citations of biomedical literature from MEDLINE, life science journals, and online books. Medical Subject Headings (MeSH), the National Library of Medicine’s controlled vocabulary thesaurus used for indexing articles for PubMed, and text words related to neglected diseases were used in order to retrieve relevant literature. The first step in this search was to determine whether the word was covered by the MeSH database. If not, the word would be searched as a text word. Although “neglected disease” was included in MeSH by PubMed as of June 10, 2015, when the literature retrieval was conducted, all disease names were also included in the search for comprehensiveness. The neglected diseases defined in this research complied with the definition from G-FINDER. The G-FINDER survey, which is conducted by Policy Cures and funded by the Bill & Melinda Gates Foundation, tracks global public, private, and philanthropic investment into product R&D for neglected diseases. Policy Cures uses a three-step filter to determine the scope of neglected diseases in G-FINDER survey: (1) the disease disproportionately affects people in developing countries; (2) there is a need for new products; (3) there is market failure. If the disease meets all three conditions, then it will be covered by the G-FINDER survey. As of the date of literature retrieval, there were 14 kinds of disease. “Research and development” was not yet added to MeSH database, and thus “research” as MeSH and “research and development” as text words were used for the search. “Drug therapy”, “diagnosis” and “vaccines” were used for the search since the study focuses on products for diagnosis, prevention and treatment.

Initially, the search was conducted without restriction on publication date or first author’s country so as to find the global trend and countries with large scientific production. Then the search was done for BRICS countries one by one with date restriction. Since articles published in 2014 were being added into the PubMed database at the time, co-occurrence cluster analysis based on high frequency subject words were conducted only for published literature prior to the end of 2013 to avoid uncertain influence from literature that had not yet been included in the database. The literature retrieval strategy for Brazil as an example is as follows:

(“Neglected Diseases”[Mesh] OR “HIV”[Mesh] OR “Malaria”[Mesh] OR “Tuberculosis”[Mesh] OR “Chagas Disease”[Mesh] OR “Leishmaniasis”[Mesh] OR “Trypanosomiasis, African”[Mesh] OR “Diarrhea”[Mesh] OR “Salmonella Infections”[Mesh] OR “Dengue”[Mesh] OR “Pneumonia, Bacterial”[Mesh] OR “Meningitis, Bacterial”[Mesh] OR “Leprosy”[Mesh] OR “Buruli Ulcer”[Mesh] OR “Trachoma”[Mesh] OR “Rheumatic Fever”[Mesh] OR “Helminths”[Mesh]) AND (“Research and Development”[Text Word] OR “development and research”[Text Word] OR “Research”[Mesh] OR “Research”[Text Word]) AND (“drug therapy”[Mesh] OR “diagnosis”[Mesh] OR “Vaccines”[Mesh]) AND “Brazil” [Affiliation] AND (“0001/01/01”[PDAT] : “2013/12/31”[PDAT]).

Each copy of a paper set for each country in XML format was downloaded. BICOMS 2.0 and gCLUTO 1.0 were used to conduct the bibliometric analysis, specifically, the co-occurrence of high frequency subject words. BICOMS can accurately extract and count the bibliographic information from worldwide databases to generate a co-occurrence matrix and provide basic data for subsequent statistical analysis. The gCLUTO is interactive software for clustering low- and high-dimensional datasets and analyzing the characteristics of the various clusters. Subject words are the main information source for obtaining the internal characteristics of papers, and the co-occurrence can divide subject words into different categories in a way in which the research hotspots can be reflected. Subject words were extracted from the paper set through “major subject heading - subheading” in BICOMS and the extracted words were ranked based on their frequency in each paper set. High frequency words with the cumulative percentage of over 30 % were extracted to construct a word-article matrix. If the number of high frequency words for the matrix was less than 30, then subject words with cumulative percent over 40 % or even 50 % were extracted until the word number exceeded 30. Then the word-article matrix was used to conduct co-occurrence double clustering analysis with gCLUTO, selecting “repeat dichotomy”, “cosine” in “similar function” and “I2” in “criterion function”. Clustering results with the highest average Isim (similarity in intra-class) and lowest average Esim (similarity in inter-class) were defined as optimal results, from which a double clustering hill diagram was built. A double clustering hill diagram was also used to roughly estimate the clustering effect. Usually, different colors can appear in hills, including red, yellow, green, light blue and dark blue. The color of the peak is meaningful, as red represents low standard deviation while dark blue represents high standard deviation. The height of the hill is proportional to the similarity in intra-class and the volume of the hill is proportional to the amount of articles. Three articles with high description and discrimination of each cluster that carried the core information were provided by gCLUTO. A dendrogram was also produced to show the clusters of high frequency subject words. The abstracts of three high descriptive articles and high frequency subject words for each cluster were used to identify hotspots. A hotspot in the text corresponds to one in the hill diagram. The search strategy was also used in GoPubMed for complementary interpretation of the search results and clustering.

## Result

**Fig. 1:**
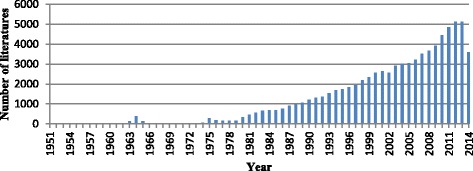
Global literature about R&D on neglected diseases published from 1951 to 2014

A total of 80 625 records were obtained up to June 10, 2015. The earliest articles were published in 1951. The amount of literature began to grow rapidly and steadily starting from 1975, though a short-term increase was observed from 1963 to 1965 (see Fig. 1). Articles published in 2014 were being added into the database of PubMed when the search was conducted and thus its number is much less than the number of articles from 2013.

**Fig. 2:**
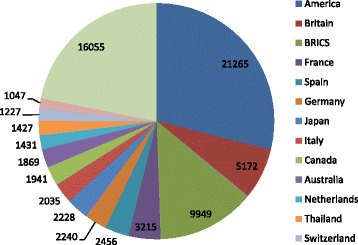
Literature about R&D on neglected diseases by country

Ten countries with the largest amount of published literature on R&D for neglected diseases are America, Britain, Brazil, France, Spain, China, India, Germany, Japan and Italy. America issued 2 1265 articles, accounting for 26.38 % of the world’s literature, followed by Britain’s 5172. Among BRICS, Brazil (3 649) and Russia (225) are countries with the largest and smallest literature amount respectively. The number of articles from India, China and South Africa is 2 353, 2 417 and 1 305 respectively. A total of 9 949 articles are from BRICS countries, accounting for 12.34 % of the world’s literature (see Fig. 2).

The first article from the BRICS was published in 1984 and the first author of this article was from India. The article focuses on Bacillus Calmette-Guérin (BCG) vaccination’s protection against the “adult form” of tuberculosis [10]. The first articles from Brazil, Russia, China and South Africa were published in 1987, 1993, 1988 and 1987 respectively. In total, 17 articles published in 1987 from Brazil were collected by PubMed and they are about chagas disease [11], schistosoma mansoni [12] and leishmaniasis [13]. The earliest two articles from Russia are about vaccines for HIV-1 [14] and tuberculosis [15]. The first articles from China and South Africa are on neutralizing antibodies against natural infections with human rotavirus [16] and the diagnosis of hepatic tuberculosis respectively [17].

**Fig. 3:**
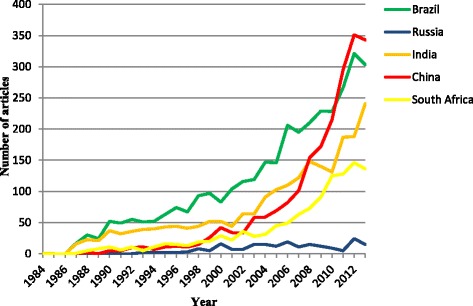
Annual published articles about R&D on neglected diseases for BRICS from 1984 to 2013

Though the first article from BRICS was published by India, Brazil ranked first in terms of the annual amount of published articles until 2011 when it was surpassed by China. As to the growth in annual number of published articles, Brazil has been increasing rapidly the entire time. It seems that Russia tends to reach a plateau and the number of annual published articles has been below 25. The number of articles published annually by India has been increasing quickly with the exception of relatively slow growth during the period from 1994 to 2003. The number of articles from China soared since 2005 while South Africa underwent a rapid growth period after 2007 (see Fig. 3). The volume of articles from the BRICS in 2013 were less than in 2012, but according to “Relative Research Interest” from GoPubMed, the research interest of BRICS countries, except Russia, on neglected diseases still grows.

The amount of literature included in the paper sets from Brazil, Russia, India, China and South Africa for high frequency subject words extraction is 2 730, 162, 2 029, 2 199 and 1 131 respectively. According to the inclusion criteria for clustering analysis, high frequency subject words extracted using BICOMS number 32 (cumulative percent: 31.78 %), 47 (51.99 %), 51 (40.56 %), 31 (31.79 %) and 44 (50.52 %) for the BRICS.

**Fig. 4:**
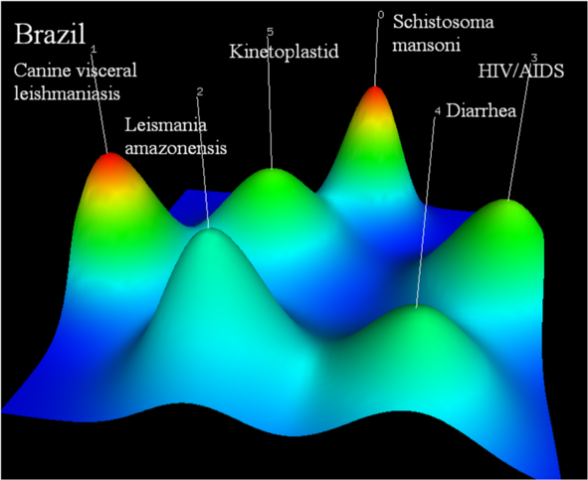
Co-occurrence clustering hill diagram of the top high-frequency subjects for Brazil

The six research hotspots in Brazil include (0) cytokine response of schistosoma mansoni infected patients: cytokine response caused by vaccine candidate and role of cytokines in hepatic fibrosis in human schistosomiasis mansoni (PMID18565118, PMID18550021, PMID15155645); (1) canine visceral leishmaniasis: whether balance between the branches of immune response and the intracellular iron availability will influence the course of leishmania infection, effectiveness of transmission blocking vaccine and the protective immunity of recombinant A2 protein (PMID24146743, PMID16386824, PMID18786587); (2) treatment of leishmania amazonensis (PMID16343984, PMID20713665, PMID 20863436); (3) treatment of HIV/AIDS: drug therapy for HIV/TB patients and antiretroviral resistance in patients presenting therapeutic failure (PMID15476053, PMID20428637, PMID17992369); (4) diarrhea: identification of escherichia coli strains by polymerase chain reaction (PCR) (PMID9511828, PMID7615758, PMID12732425); and (5) human T cell responsiveness to kinoplasmosome, such as chagas’ disease and leishmania (PMID2108932, PMID2004486, PMID3129512) (see Fig. 4).

**Fig. 5:**
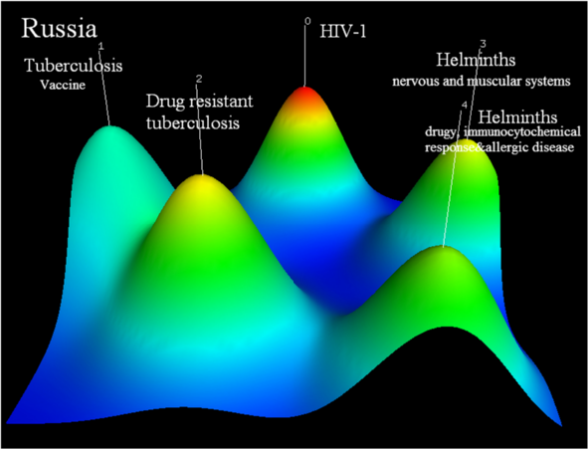
Co-occurrence clustering hill diagram of the top high-frequency subjects for Russia

Russia’s five research hotspots are (0) vaccine against HIV-1(PMID11251384, PMID15253564, PMID15068850); (1) vaccine against tuberculosis (PMID21419703, PMID 12874362, PMID16415170); (2) multidrug-resistant tuberculosis (MDR-TB): the clinical characteristics, detection of second-line drug resistance and standardized therapy development of MDR-TB patients, and association of specific gene mutations with MDR-TB (PMID15666160, PMID17403134, PMID23705640); (3) nervous and muscular systems of helminth (PMID22941527, PMID12270263, PMID18802724); and (4) helminth, including novel drug discovery and immunocytochemical response and allergic disease caused by it (PMID18652395, PMID17549516, PMID20701571) (see Fig. 5).

**Fig. 6:**
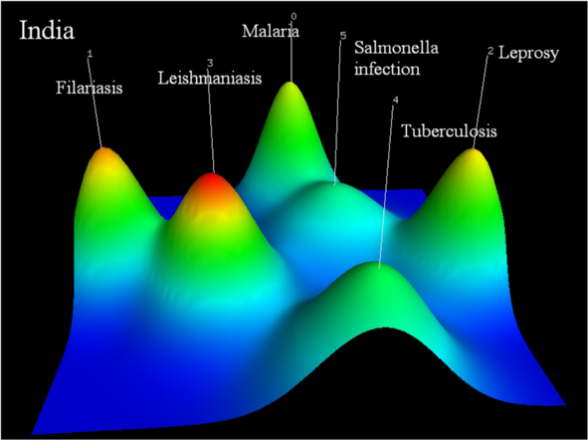
Co-occurrence clustering hill diagram of the top high-frequency subjects for India

Indian research foci include (0) mass drug administration and diagnosis of filariasis (PMID14613632, PMID21601901, PMID8758067); (1) malaria: the protection of duffy binding-like domains that bind chondroitin sulfate A against pregnancy associated malaria, immune response to plasmodium falciparum and effectiveness of extracts of plants used in the traditional medicine (PMID16988275, PMID12540535, PMID11693874); (2) vaccine against leprosy (PMID10575405, PMID10575404, PMID2659699); (3) diagnosis, treatment and vaccination of leishmania (PMID12559811, PMID20695748, PMID15364110); (4) rapid detection of mycobacteria and drug-resistance for pulmonary tuberculosis patients (PMID17350204, PMID22461679, PMID15956385); and (5) diagnosis and pathogenesis of salmonella infection (PMID2068738, PMID19520723, PMID1401194) (see Fig. 6).

**Fig. 7:**
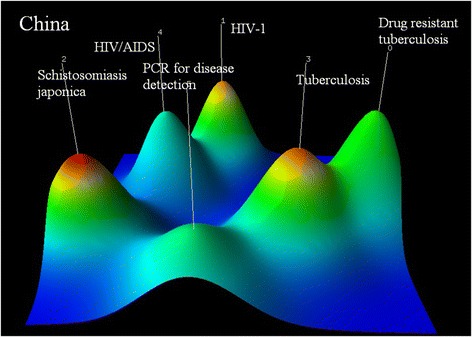
Co-occurrence clustering hill diagram of the top high-frequency subjects for China

China focuses on six areas including (0) drug-resistant tuberculosis: genotypes detection and drug susceptibility test of mycobacterium tuberculosis (PMID21392442, PMID22713520, PMID21276311); (1) vaccine against HIV-1(PMID21807049, PMID12018459, PMID10803497); (2) vaccine against schistosoma japonicum (PMID15115073, PMID17182036, PMID19812258); (3) tuberculosis: potential protection of recombinant BCG and diagnosis with gamma interferon (PMID17919299, PMID20943878, PMID19279170); (4) highly active antiretroviral therapy (HAART): dynamics of T cell response and recognition of favorite timing for initiation of the therapy (PMID16900640, PMID20108767, PMID12734930); and (5) the utilization of gene sequencing in the screening and diagnosis of disease: macrolide-resistant mycoplasma pneumonia infection, dengue and rifampin-resistant mycobacterium tuberculosis (PMID23720793, PMID19018028, PMID15472290) (see Fig. 7).

**Fig. 8:**
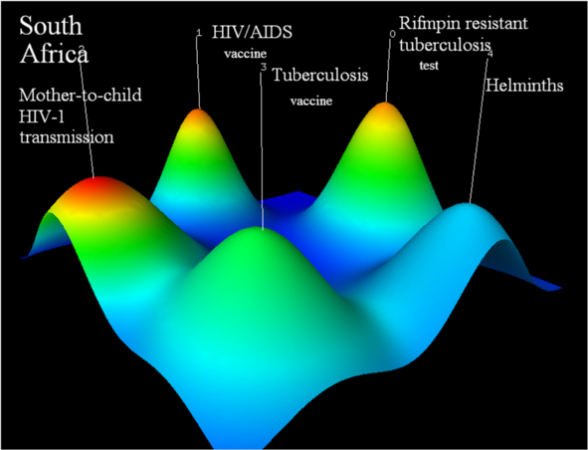
Co-occurrence clustering hill diagram of the top high-frequency subjects for South Africa

The research priorities of South Africa are (0) rapid screening test for rifampin-resistant tuberculosis and the resistance among children (PMID17985057, PMID22236850, PMID24478501); (1) prevention and protection of vaccine against HIV (PMID22106990, PMID14522014^,^ PMID22549090); (2) safety and effectiveness of nevirapine regimen to reduce mother-to-child HIV transmission (PMID16052084, PMID22196945, PMID12599045); (3) vaccine against tuberculosis that can induce CD4+ T cells response (PMID20017188, PMID23737382, PMID20224065); (4)helminth: anthelmintic effects of plant extracts and management interventions on helminth for animals (PMID16239113, PMID12053959, PMID20542105) (see Fig. 8).

Eight diseases were found to be BRICS’ research hotspots and almost all five countries make helminth, HIV/AIDS and tuberculosis their R&D priority. Although India does not include HIV/AIDS as their focus, it has the most extensive research hotspots among the five countries. In addition to helminth and tuberculosis, India also focuses on leishmania, malaria, leprosy and salmonella and the last three are only included in Indian’s hotspots. Brazil is the sole country to make diarrhea an R&D priority (see Table 1).

**Table 1:**
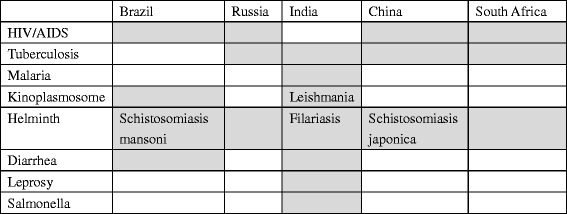
Prioritized diseases in the BRICS

Notes: The shaded cell means that a certain disease was found to be a research hotspot of the country and shaded cell with a disease name in it stands for that the specific disease type was the research focus

## Discussion

Developed countries seem to play a leading role in R&D for neglected diseases and the literature quantity is closely related to the investment. The amount of literature from America itself accounts for over 1/4 of the literature published worldwide, and is more than doubled the amount of literature from the BRICS. G-FIDER’s report shows that 3.2 billion US dollars was dedicated to neglected diseases in 2012, 63.2 % of all funding was from the public sector. About 95.9 % of public sector funds are from high-income countries (the first three are America, Britain and European Commission) and the funding from low- and middle-income countries (LMIC) is growing, though the amount is still very small. Among the five countries, only India and Brazil are on the list of top 12 funders and comes in seventh and eighth respectively [18]. Funding from India and Brazil totals 34.4 and 19.7 million US dollars (adjusted to 2007 US dollars) respectively, accounting for 1.7 % and 1.0 % of the total funding from the public sector. Funding from India, Brazil and South Africa account for almost 3/4 of the total funding from LMICs in 2013 [19]. China also published a lot of relevant literature, but its financial contribution seems quite small based on the G-FINDER report. The most probable cause might be incomplete information collection, as the data for China only covers funding for a few diseases, such as HIV/AIDS and helminthiasis.

With economic development and growing attention to healthcare, the investment from BRICS countries into R&D for neglected diseases is rising, as shown by the increased scientific production. According to the database of the Word Bank, the gross domestic product (GDP) of the BRICS has been on a stable rise since 1980 [5]. The health expenditure per capita of the five countries has also been increasing consistently since 1995 [20]. The eight Millennium Development Goals (MDG) put forth in 2000 also push governments to act to improve health status, and of which the MDG 6 is regarding HIV/AIDS, malaria and tuberculosis. *Public Health Innovation and Intellectual Property Rights* presented at the 56^th^ World Health Assembly noted that R&D in the pharmaceutical sector must address public health needs and not only potential market gains [21]. WHA61.21 was adopted to implement the Global Strategy and Plan of Action on Public Health, Innovation and Intellectual Property and member states agreed in 2008 to promote new thinking on innovation and access to medicines, as well as establish an enhanced and sustainable basis for need-driven essential health R&D relevant to diseases which disproportionately affect developing countries [22]. Our study also finds a rapid increase in literature since the beginning of 21^st^ century, which might be due to the attention to health and neglected diseases from the international community.

Unlike the global research focus, BRICS countries tend to focus on major causes of disease burden among the neglected diseases. Globally, HIV/AIDS is the top priority among neglected diseases and the amount of literature on its R&D is much larger than that of the following parasitic disease (especially malaria) and tuberculosis. G-FINDER survey also reports that HIV/AIDS, malaria and tuberculosis receive the vast majority of global neglected disease R&D funding [18]. It seems that the BRICS research hotspots are related to their disease burdens. According to data on the WHO’s Global Burden of Disease in 2012 [23], the first three causes among neglected diseases for disability-adjusted life-year (DALY) in BRICS countries include HIV/AIDS, parasitic and vector diseases, diarrhea and tuberculosis, which are exactly the hotspots found in the study. In total, 30 % of the children at risk of soil transmitted helminthiasis around the world are from the BRICS [24] and thus it is not strange that helminthiasis is an R&D priority for the five countries. Except for Russia and South Africa; Brazil, India and China focus on one specific helminthiasis type which is highly consistent with their disease burden. Indian residents at risk of filariasis infection account for 50 % of the total population at risk, and filariasis is treated as a research focus by India [25]. Brazil and China both make schistosomiasis a priority, but they only attach importance to a specific epidemic type in their own country. The absolute number of leprosy patients in India is 87 000, which seems not to be very large, but it accounts for 41 % the global patients [26] and thus it is reasonable that leprosy is found as one hotspot. Russia has the least burden of neglected diseases among the five and the major cause for its DALY is chronic disease. Our research also finds the amount of Russia’s literature is much less than the others. New drugs developed by Russian enterprises are usually only put on the domestic market, which might also affect the literature publishing [7]. Since the research hotspots in the BRICS countries are not completely the same, they would benefit from each other for exchanging experience in the common research areas while sharing R&D results for research foci peculiar to one country. Thus, BRICS countries can work together to better utilize R&D resources and cure diseases that affect developing countries.

However, disease burden is not the only factor influencing the research hotspots. It is very interesting that HIV/AIDS is not one of the Indian R&D hotspots even though the disease burden is very heavy [23]. The possible reason might be the Indian patent law, which allows for generic production of drugs under certain conditions. According to the law, Indian pharmaceutical factories are only allowed to continue the generic production of drugs brought to market before 1995. As for drugs introduced to India from 1995 to 2005, generic manufacturers can produce them as long as they pay “reasonable royalties” to the patent holders. Drugs introduced after 2005 can be generically produced only after the patents expire (last for 20 years). However, compulsory licensing can be used by India if necessary since the WTO’s decision of 30 August 2003 (Article 31bis of TRIPS) expands the potential use of compulsory licensing to allow the manufacture of generic versions of patented drugs for exports to least-developed countries [27, 28]. Most anti-retroviral drugs were introduced prior to 2005 and thus currently India is a major supplier of affordable generic anti-retroviral medicines to developing countries, which can bring in substantial profit. Besides, unlike other neglected disease that usually confined in certain areas, HIV/AIDS is a global issue. Almost all countries, especially developed countries, pay close attention to R&D for HIV related products. In this case, India can have a free ride as the country can issue compulsory licenses to supply domestic markets or to produce for export, and India has experience in compulsory licensing [29].

As the results shown, a substantial majority of global studies as well as the investment were focused on HIV/AIDS, tuberculosis and malaria. China should conduct more studies on other neglected diseases for long-term benefits, not just three diseases that currently have gained close attention. In addition, China should not only focus on the major causes of disease burden for its own country, but also the diseases that mainly affect other developing countries. As the largest developing country, China should shoulder more responsibility in aiding other developing countries.

### Limitations

The majority of journals and literature included in PubMed are in English while English is not the mother tongue for some BRICS countries and thus this study may not comprehensively landscape them. The non-inclusion of all national journals may also influence the result to some degree. Based on the search through CNKI (China National Knowledge Infrastructure), a comprehensive system of China’s academic knowledge resources, the earliest records on treatment or diagnosis of schistosomiasis and tuberculosis can be traced back to 1950. However, the literature is about transferring foreign technology into China instead of independent R&D, thus literature in PubMed probably includes all the cutting-edge and key research of each country. Besides, hotspots clustered based on published literature stands to be the key research focus; not R&D capacity. For example, China has made great achievements in drug R&D for malaria, but malaria is not found to be a hotspot and thus over-interpretation should be avoided.

## Conclusions

Up to now, developed countries still play the key role in R&D for neglected diseases that mainly afflict the population of developing countries. However, the funding and quantity of research from the BRICS, the innovative developing counties, are growing. In contrast to the global landscape, BRICS countries focus on the major causes of disease burden for their own country and thus their research hotspots do not completely overlap. However, the disease burden is not the only determinant of the hotspots; the domestic policy, especially patent law, may also exert influence on research focus.

## Additional file

Additional file 1: Multilingual abstracts in the five official working languages of the United Nations. (PDF 185 kb)

## Abbreviations

BCG: Bacillus Calmette-Guérin
BICOMS: Bibliography Item Co-occurrence Mining System
BRICS: Brazil, Russia, India, China and South Africa
CNKI: China National Knowledge Infrastructure
DALY: Disability-adjusted life-year
gCLUTO: Graphical Clustering Toolkit
GDP: Gross domestic product
G-FINDER: Global Funding of Innovation for Neglected Disease
HAART: Highly active antiretroviral therapy
HIV/AIDS: Human immunodeficiency virus infection and acquired immune deficiency syndrome
LMIC: Low- and middle-income countries
MDG: Millennium Development Goals
MDR-TB: Multidrug-resistant tuberculosis
MeSH: Medical Subject Headings
PCR: Polymerase chain reaction
R&D: Research and development
WHO: World Health Organization
