# Supplementary material for: Bibliometric study of research and development for neglected diseases in the BRICS
Source: Infect Dis Poverty. 2016 Sep 6;5(1):89. doi: 10.1186/s40249-016-0182-1 (PMC5011792; doi:10.1186/s40249-016-0182-1)
Supplement: Additional file 1: — Multilingual abstracts in the five official working languages of the United Nations. (PDF 185 kb) [file 40249_2016_182_MOESM1_ESM.pdf]

Translation of the abstract into the five official working languages of the United Nations

دراسة ببليومترية للبحث والتطوير في مجال الأمراض المهملة في بلدان البرازيل وروسيا والهند والصين وجنوب أفريقيا (بريكس)

جينج باي، وى لى ، يانج مو هوانج ويان جوه

الملخص

الخلفية: تعاني أعداد كبيرة من الناس من مجموعة من الأمراض التي تؤثر بشكل رئيسي على البلدان النامية، حيث لا توجد منتجات متاحة أو معقولة التكلفة للوقاية أو العلاج. ولا يزال البحث والتطوير (R & D) لهذه الأمراض ذا أولوية منخفضة على جدول أعمال الصحة. يوجد في البرازيل وروسيا والهند والصين وجنوب افريقيا (بريكس) اقتصاد متزايد السرعة وله تأثير أكثر وأكثر إيجابية على الصحة العالمية. بالإضافة إلى ذلك، يُعْتَقَدْ أن قدرتهم على البحث والتطوير R & D سيتم تعزيزها من خلال عقود من الاستثمار في التعليم وبحوث علوم الحياة. ومن المتوقع أن هذه الدول (بريكس)، كمجموعة من الدول الناشئة والنامية، ستقدم مساهمات أكبر في حل المشكلة التي تؤثر بشكل رئيسي على مجتمع البلدان النامية بأكملها. ومع ذلك، كان هناك القليل من البحث لتقديم لمحة عيانية عن جهود البريكس في مجال البحث والتطوير للأمراض المهملة. والهدف من هذه الدراسة هو بحث الإنتاج العلمي لدول البريكس في هذا المجال، ونقاط البحوث الساخنة الرئيسية.

الطرق: تم فحص مجموع ما كتب عالمياً ذي الصلة دون حدود زمنية من خلال PubMed وتم تحديد البلدان ذات الحصيلة العالية باستخدام GoPubMed. تم الحصول على كل ما كتب حتى نهاية عام 2013 من دول البريكس واستخلصت الكلمات ذات التكرار العالي وتم تجميعها باستخدام مراجع نظام تنقيب توارد البند 2.0 (BICOMS) وأدوات تجميع الرسومات 1.0 (gCLUTO).

النتائج: في المجموع، تم استخلاص على التوالي 32، 47، 51، 31، 44 كلمة ذات تردد عالٍ من البرازيل وروسيا والهند والصين وجنوب أفريقيا لتحليل التجميع. وأشار التجميع إلى أن ثمانية أمراض كانت نقاط بحوث ساخنة في دول البريكس. وكان للهند النقاط الساخنة الأكثر انتشاراً وجاءت البرازيل في المركز الثاني. وتقاسمت ثلاثة بلدان أخرى بؤر بحث مشتركة: الديدان الطفيلية، عدوى فيروس نقص المناعة البشرية ومتلازمة نقص المناعة المكتسب (HIV / AIDS) والسل.

الاستنتاجات: لا تزال الدول المتقدمة تقدم غالبية المساهمات للبحث والتطوير R & D في الأمراض المهملة، ولكن دول البريكس تلعب دوراً متزايداً. فبدلاً من "الثلاثة أمراض الكبيرة" (فيروس نقص المناعة البشرية/الإيدز والملاريا والسل) المعترف بها من قِبَل منظمة الصحة العالمية، تركز دول البريكس أكثر على الأسباب الرئيسية لعبء المرض في بلدانهم. ويضع عبء المرض والسياسة الداخلية، وخصوصاً قانون براءات الاختراع، تأثيراً أساسياً على تركيز البحوث.

Translated from English version into Arabic by Free bird, through

TRANSLATORS
WITHOUT BORDERS

金砖国家被忽视疾病研发文献计量学研究

白婧，李维，黄旸木，郭岩

摘要

引言：由于缺乏诊疗和预防产品或现有产品价格不可及，被忽视疾病一直影响着发展中国家人口的健康。被忽视疾病的研发也一直未纳入卫生议程的重点。另一方面，巴西、俄罗斯、印度、中国和南非作为快速发展的经济体正对全球卫生产生越来越积极的影响。另外，近几

十年对生命科学研究的投入使得金砖国家的研发能力得到了增强。同时国际社会也期待金砖国家作为正在崛起和发展的国家能够对解决影响发展中国家的问题做出更多的贡献。然而，目前几乎没有针对金砖国家被忽视疾病研发领域整体概况的研究。因此本研究主要探索金砖国家在被忽视疾病研发的科研产出和研究热点。
方法：通过利用 PubMed 和 GoPubMed 文摘索引的结果分析相关文献的年代和国家分布，并利用 PubMed 检索金砖国家截至 2013 年年底发表的相关文献。利用书目共现分析系统（BICOMS）统计金砖国家被忽视疾病研发研究主题词出现的频次，利用 gCLUTO 软件对高频主题词进行共现分析。
结果：巴西、俄罗斯、印度、中国和南纳入共现分析的主题词分别有 32、47、51、31 和 44 个。聚类结果显示金砖国家研究热点主要集中在 8 种疾病。印度的研究热点最为广泛，巴西其次为巴西。其他三个国家主要关注蠕虫病、HIV/AIDS 和结核病。
结论：在被忽视疾病研发领域，仍然是发达国家在发挥主要作用，但是金砖国家正在发挥着越来越重要的作用。相对于“三大疾病”（HIV/AIDS、疟疾和结核病而言，金砖国家更关注主要影响本国的被忽视疾病。疾病负担、国内政策，尤其是专利法对金砖国家被忽视疾病的研究热点具有重要影响。

Translated from English version into Chinese by Bai Jing

Étude bibliométrique de l'activité de recherche et développement liée aux maladies négligées dans les BRICS

Jing Bai, Wei Li, Yang-Mu Huang et Yan Guo

Résumé
Contexte : Un groupe de maladies touche de larges populations, qui sont principalement situées dans les pays en développement en raison de l'absence de moyens de prévention et de traitement disponibles ou abordables. L'agenda sanitaire n'accorde qu'une faible priorité à l'activité de recherche et développement (R&D) liée à ces maladies. Le Brésil, la Russie, l'Inde, la Chine et l'Afrique du Sud (les BRICS) sont des économies connaissant une croissance rapide, dont l'impact sur la santé au niveau mondial est de plus en plus positif. En outre, on estime que leur capacité de R&D est accrue par des décennies d'investissement dans l'éducation et la recherche en sciences de la vie. On s'attend à ce que les BRICS, en tant que groupe de pays émergents et en développement, contribuent davantage à résoudre ce problème affectant principalement l'ensemble de la communauté des pays en développement. Cependant, peu de recherches ont été effectuées qui fournissent un aperçu macroscopique des efforts de R&D des BRICS en matière de maladies négligées. Cette étude vise à examiner la production scientifique des BRICS dans ce domaine, ainsi que les principaux secteurs concentrant leurs recherches.
Méthodes : La littérature mondiale pertinente a fait l'objet d'une recherche sans limite de temps par le biais de PubMed et les pays à haute production ont été identifiés à l'aide de GoPubMed. Après obtention de la littérature en provenance des BRICS publiée jusqu'à fin 2013, les mots à haute fréquence en ont été extraits et agglomérés à l'aide de Bibliography Item Co-occurrence Mining System 2.0 (BICOMS) et de Graphical Clustering Toolkit 1.0 (gCLUTO).
Résultats : Au total, 32, 47, 51, 31 et 44 mots à haute fréquence ont été extraits de la littérature

publiée respectivement au Brésil, en Russie, en Inde, en Chine et en Afrique du Sud à des fins d'analyse par segmentation. Selon cette segmentation, les recherches effectuées dans les BRICS se concentrent sur huit maladies. L'Inde présente les plus vastes secteurs de recherche, suivie du Brésil. Les recherches effectuées dans les trois autres pays se concentrent sur les mêmes domaines : l'helminthiase, les virus de l'immunodéficience humaine et syndrome d'immunodéficience acquise (VIH/SIDA) et la tuberculose.
Conclusions : Si les pays développés effectuent toujours la majorité des contributions en matière de R&D liée aux maladies négligées, les BRICS y jouent un rôle croissant. Au lieu des « trois grandes maladies » reconnues par l'OMS (le VIH/SIDA, le paludisme et la tuberculose), les BRICS se concentrent plutôt sur les principales causes de la charge de morbidité qu'ils enregistrent. Le domaine de recherche est principalement influencé par la charge de morbidité et la politique intérieure, en particulier la loi sur les brevets.

Translated from English version into French by Clémentine Choubrac, through

TRANSLATORS WITHOUT BORDERS

Библиометрический анализ научно-исследовательской деятельности в отношении «забытых» заболеваний в странах BRICS

Джинг Баи, Вэй Ли, Янг-Му Хуанг и Ян Гуо

Резюме
Краткая информация: Большое количество людей страдает заболеваниями, характерными для развивающихся стран, вследствие отсутствия доступа к средствам для их профилактики и лечения. Научные исследования данных заболеваний занимают второстепенное место в перечне основных мероприятий по охране здоровья. Бразилия, Россия, Индия, Китай и Южная Африка (BRICS) являются не только странами с быстроразвивающимися экономиками, но и оказывают все больше позитивного влияния на мировую систему охраны здоровья. Кроме того, их научно-исследовательский потенциал обусловлен десятилетиями инвестиций в образование и медико-биологические исследования. Будучи растущими и развивающимися государствами, страны BRICS, теоретически, должны делать большие вложения в решение проблем, с которыми сталкивается преимущественно население развивающихся стран. Однако, детальных исследований в области усилий стран BRICS по научным исследованиям в области так называемых забытых заболеваний слишком мало. Цель данного исследования – анализ научных изысканий в указанной области и основные статьи соответствующего поиска в странах BRICS.
Методы: Установлено, что поиск соответствующей всемирной литературы производился непрерывно через поисковую систему PubMed, а в странах с высокими доходами - через GoPubMed. Была проанализирована литература стран BRICS до конца 2013 года, и выделены и сгруппированы наиболее часто встречающиеся слова поиска с использованием

системы BibliographyItemCo-occurrenceMiningSystem 2.0 (BICOMS) и приложения GraphicalClusteringToolkit 1.0 (gCLUTO).

Результаты: Всего 32, 47, 51, 31 и 44 часто встречающихся слова из Бразилии, России, Индии, Китая и Южной Африки соответственно были выделены для проведения кластерного анализа. По результатам кластеризации, в странах BRICS лидирует по поисковым запросам 8 заболеваний. Индия занимает первое место по широте поиска, на втором месте – Бразилия. В остальных трех странах наиболее часто встречающимися статьями поиска оказались: глистная инвазия, вирус иммунодефицита человека и синдром приобретённого иммунодефицита (ВИЧ/СПИД), и туберкулез.

Выводы: Развивающиеся страны по-прежнему направляют большую часть вложений на исследования и разработки в области забытых заболеваний, при возрастающей роли стран BRICS. В отличие от ВОЗ, фокусирующей внимание на так называемой «большой тройке» (ВИЧ/СПИД, малярия и туберкулез), стран BRICS сосредоточены на основных причинах заболеваний. Бремя болезней и внутренняя политика, в особенности патентное право, оказывают первостепенное влияние на направление исследований.

Translated from English version into Russian by Ms Zhdanova, through

TRANSLATORS
WITHOUT BORDERS

Estudio bibliométrico de la investigación y el desarrollo para las enfermedades desatendidas en el BRICS

Jing Bai, Wei Li, Yang Mu-Huang y Yan Guo

Sumario

Fundamentos: Un gran número de personas están sufriendo de un grupo de enfermedades que afectan principalmente a los países en desarrollo, ya que no hay productos disponibles o asequibles para la prevención o el tratamiento. Las actividades de Investigación y Desarrollo (ID) para estas enfermedades sigue siendo una prioridad baja en la agenda sanitaria. Brasil, Rusia, India, China y Sudáfrica (BRICS) son economías en creciendo rápido crecimiento y tienen cada vez más impacto y más positivo en la salud mundial. Además, se cree que la capacidad de sus actividades de ID ha mejorado a través de décadas de inversiones en la educación y la investigación en ciencias biológicas. Se espera que el BRICS, como grupo de países emergentes y en desarrollo, haga mayores contribuciones a la solución del problema que afecta principalmente a toda la comunidad de los países en desarrollo. Sin embargo, ha habido poca investigación para proporcionar una visión macroscópica del esfuerzo del BRICS en ID para las enfermedades desatendidas. El objetivo de este estudio es investigar la producción científica de los países del BRICS en esta área y sus puntos principales de investigación.

Métodos: Se registró la literatura mundial relevante sin límites de tiempo a través de PubMed y, usando GoPubMed, se identificaron los países de alto rendimiento. Se obtuvo la literatura del BRICS hasta finales de 2013, se extrajeron las palabras de alta frecuencia y se agruparon

utilizando Bibliography Item Co-occurrence Mining System 2.0 (BICOMS) and Graphical Clustering Toolkit 1.0 (gCLUTO).

Resultados: En total, se extrajeron 32, 47, 51, 31 y 44 palabras de alta frecuencia de Brasil, Rusia, India, China y Sudáfrica, respectivamente, para análisis de agrupamiento. La agrupación indicó que ocho enfermedades eran puntos principales de investigación en los países del BRICS. La India tenía las más amplias zonas activas y Brasil quedó en segundo lugar. Los otros tres países comparten focos comunes de investigación: la helmintiasis, la inmunodeficiencia humana infección por el virus y el síndrome de inmunodeficiencia adquirida (VIH/SIDA) y la tuberculosis

Conclusiones: Los países desarrollados todavía hacen la mayoría de las contribuciones a ID en enfermedades desatendidas, pero los países del BRICS están desempeñando un papel cada vez mayor. En lugar de las "tres grandes enfermedades" (VIH/SIDA, malaria y tuberculosis) reconocidas por la OMS, el BRICS se centra más en las principales causas de la carga de la enfermedad en sus propios países. La carga de la enfermedad y de la política interna, en particular la ley de patentes, ejercen una influencia fundamental sobre el foco de la investigación.

Translated from English version into Spanish by María Diehn, through

TRANSLATORS
WITHOUT BORDERS
